# "It depends who you ask": perceptions of the family environment of adolescents presenting to a specialist eating disorders program

**DOI:** 10.1186/2050-2974-1-S1-O56

**Published:** 2013-11-14

**Authors:** Elizabeth K Hughes, Erica Allan, Daniel Le Grange, Susan Sawyer

**Affiliations:** 1 University of Melbourne, Australia; 2 Murdoch Childrens Research Institute, Australia; 3 Centre for Adolescent Health, Royal Children's Hospital, Australia; 4 University of Melbourne, Australia; 5 University of Chicago, USA

## 

Assessment of the family environment of adolescents with eating disorders is a standard part of clinical and research practice. It can identify factors that maintain symptoms or that have the potential to impede treatment progress. It can also be important for evaluating and monitoring the impact of the illness on the family and changes in family dynamics over the course of treatment.

A comprehensive assessment will typically take a multi-informant approach by obtaining reports from a number of family members. However, reports may differ markedly between informants and be difficult to interpret. Further, discrepancies can be indicative of disturbances in the family or its members.

We administered the Family Environment Scale (Cohesion, Expressiveness, Conflict) to families presenting to a specialist eating disorder program (94 adolescent-mother, 71 adolescent-father, 76 mother-father pairs). Mean scores were similar across informants, aside from slightly lower levels of cohesion and expression for adolescents than mothers and fathers respectively. Within families, however, many dyads held discrepant perspectives. Up to half of adolescent-parent dyads and a third of mother-father dyads reported significantly different scores. This presentation will further describe these discrepancies, their relation to other factors including parent and adolescent psychopathology, and implications for research and clinical practice.

This abstract was presented in the **Children and Youth Treatment and Service Development** stream of the 2013 ANZAED Conference.

